# A case report of infantile fibrosarcoma with BRAF gene mutation with incomplete intestinal obstruction

**DOI:** 10.3389/fonc.2025.1492654

**Published:** 2025-01-29

**Authors:** Fan Zhu, Liang Fan, Guanhua Cui, Hai Jian, Hongjie Zhou, Jianfeng Xu, Fengshun Chen

**Affiliations:** General Surgery (Pediatric Surgery), Huizhou First Maternal and Child Health Care Hospital, Huizhou, China

**Keywords:** ileum, infantile fibrosarcoma of the intestine, clinical and pathological features, BRAFgene mutation, incomplete intestinal obstruction

## Abstract

**Objective:**

This study aims to explore the clinical features, diagnosis, and treatment of infantile fibrosarcoma (IFS) associated with BRAF mutations, with the goal of enhancing clinicians’ understanding of this rare genetic variant and its relationship to IFS.

**Methods:**

The China National Knowledge Infrastructure (CNKI), Wanfang Database, VIP Database, PubMed, and National Center for Biotechnology Information (NCBI) were searched using the keywords “infantile fibrosarcoma” and “congenital fibrosarcoma” for relevant articles published before August 2024. A total of 529 articles and 498 cases were identified, of which 48 articles and 149 cases were in Chinese and 479 articles and 349 cases were in foreign languages. Among them, 20 cases occurred in the gastrointestinal tract, with two cases associated with BRAF gene mutation. Combining the case reported in this paper, the clinical manifestations and treatment experience were summarized.

**Results:**

The patient was a male infant aged 5 months 18 days who presented with vomiting for 4 days. Preoperative abdominal ultrasonography revealed an abnormal hyper-echoic mass in the right upper abdomen. Exploratory laparotomy and complete tumor excision were performed. Pathological examination confirmed a diagnosis of IFS, with molecular analysis identifying a BRAF p.V600delinsDL mutation. Postoperative follow-up over 8 months showed no recurrence or metastasis on abdominal ultrasound. A review of this case alongside 20 reported cases of intestinal IFS revealed that intestinal perforation and obstruction were the most common presentations, with favorable overall prognoses.

**Conclusion:**

Intestinal IFS is a rare soft tissue sarcoma predominantly occurring in early infancy. Cases involving the BRAF p.V600delinsDL mutation are even rarer. Treatment should be individualized, with complete surgical resection being the cornerstone of therapy. Prognosis remains favorable following complete excision.

## 1 Materials and methods

### 1.1 Case presentation

**Figure 1:**
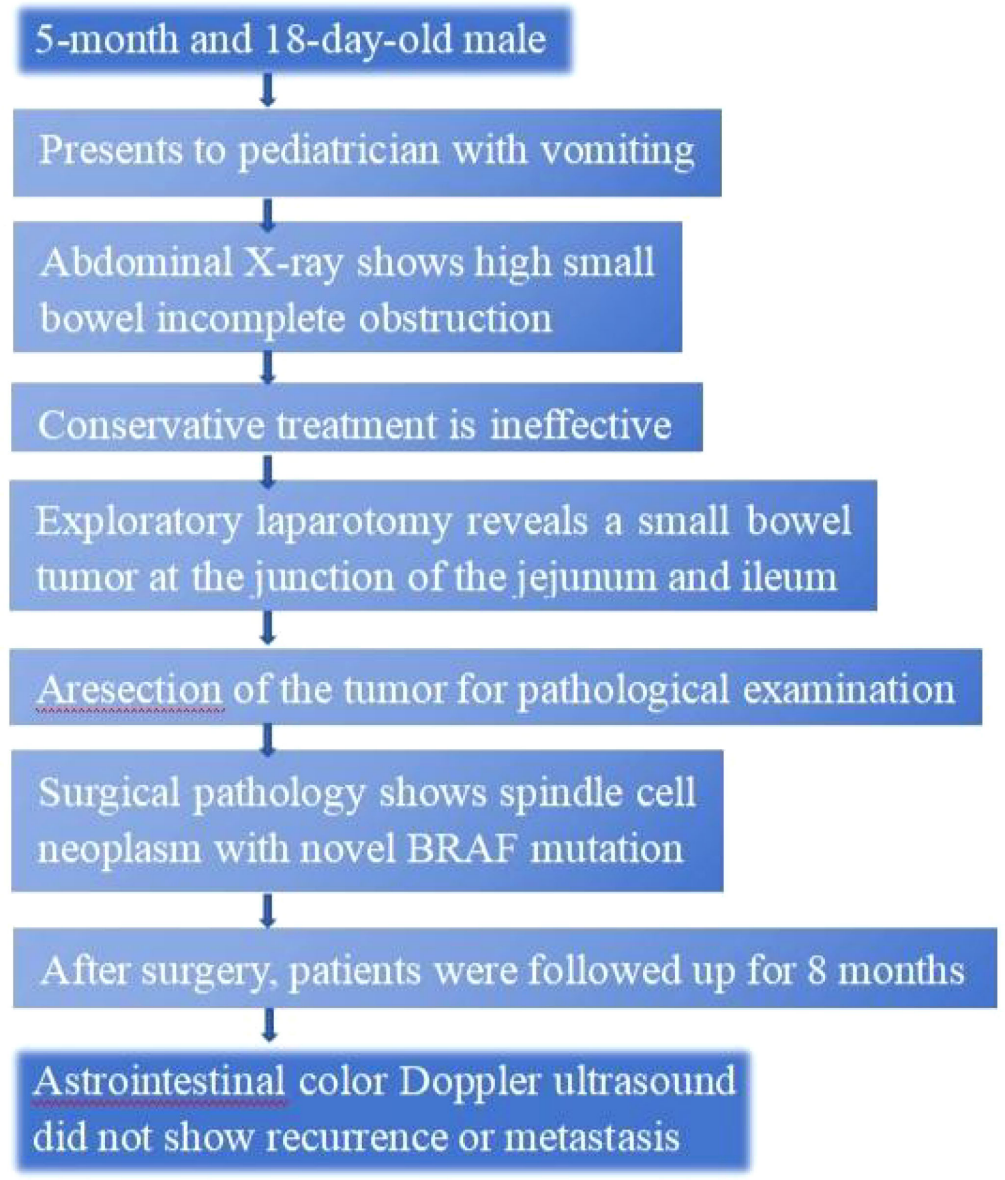
Timeline. A graphic depiction of the timeline of events in this case.

This study presents a case of a spindle cell tumor with a BRAF mutation detected through pathological examination (**Figure 1**). Its histological appearance resembles infantile fibrosarcoma (IFS) yet lacks the hallmark ETV6–NTRK3 tyrosine kinase fusion mutation commonly observed in IFS (1–3). This is the third reported case of a BRAF-mutated IFS-like tumor associated with gastrointestinal disease, as described in the literature (4, 10).

**Figure 2:**
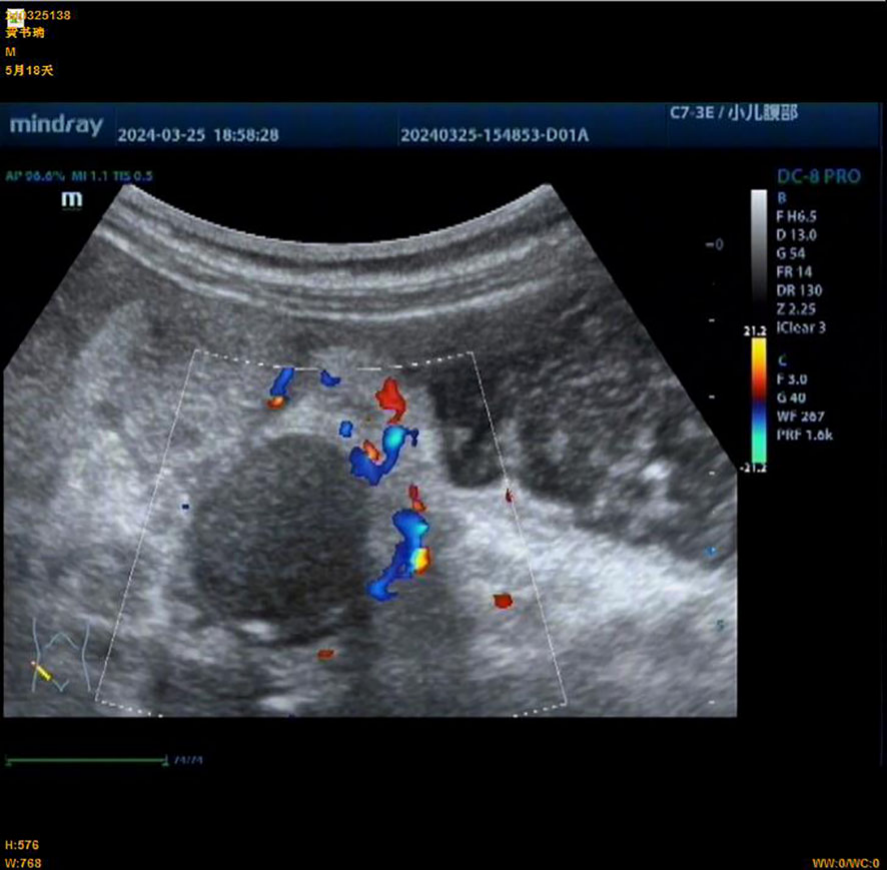
Preoperative abdominal ultrasound of an infant with congenital fibrosarcoma. An abnormal echo mass is seen in the right upper abdomen, with a range of approximately 31 × 19 mm, unclear boundaries, and uneven internal echoes.

**Figure 3:**
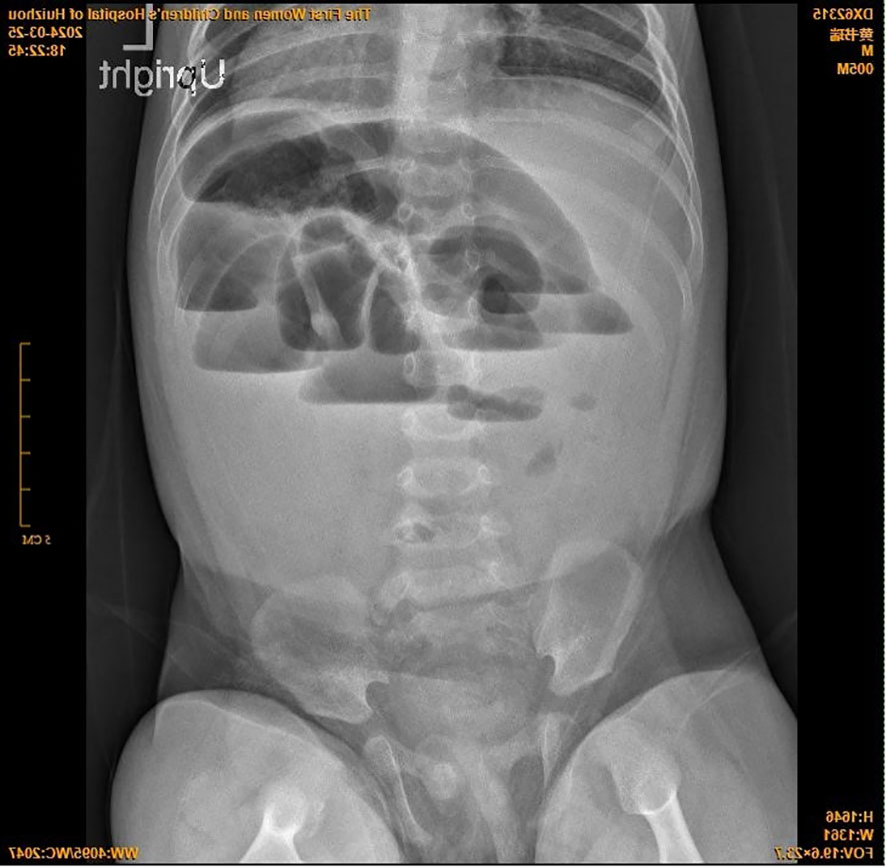
Upright abdominal plain film.

**Figure 4:**
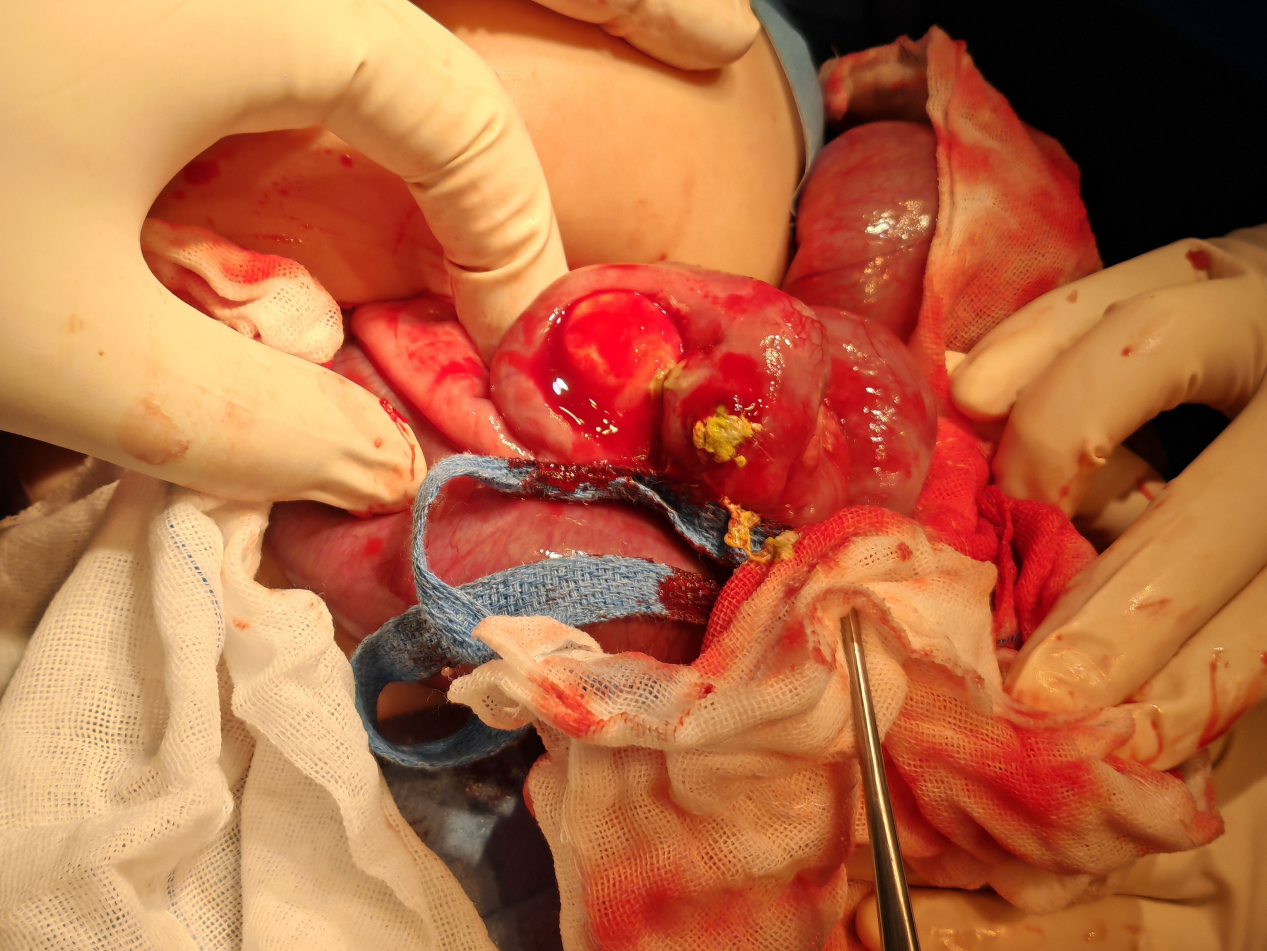
Intraoperative view of resection of tumor and diseased bowel in a patient with infantile fibrosarcoma.

**Figure 5:**
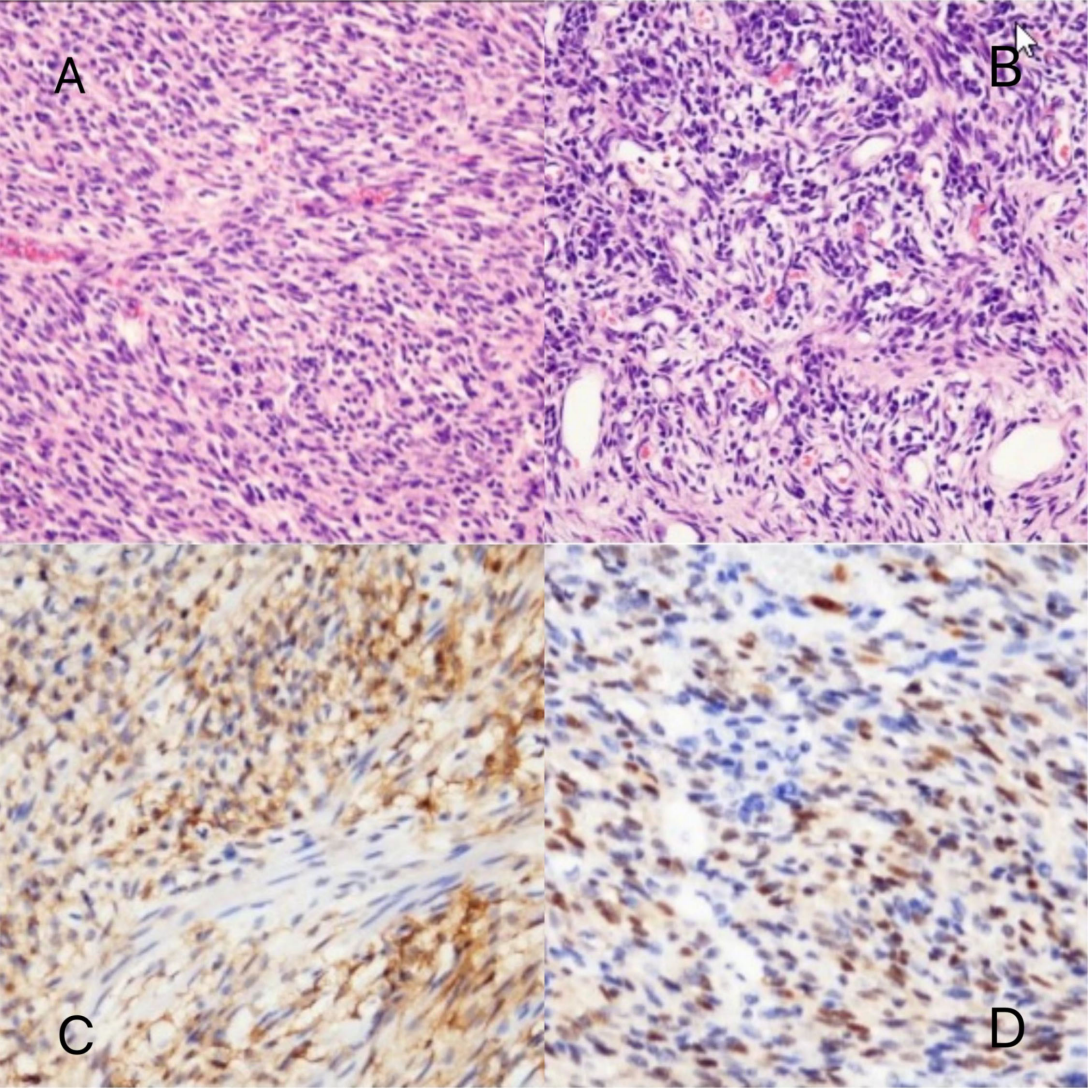
Pathomorphologic findings in a patient with infantile fibrosarcoma. **(A, B)** Dense spindle-shaped cells arranged in bundles, with dilated blood vessels visible. **(C)** CD10 partially (+). **(D)** Cyclin D1 (partially +).

**Figure 6:**
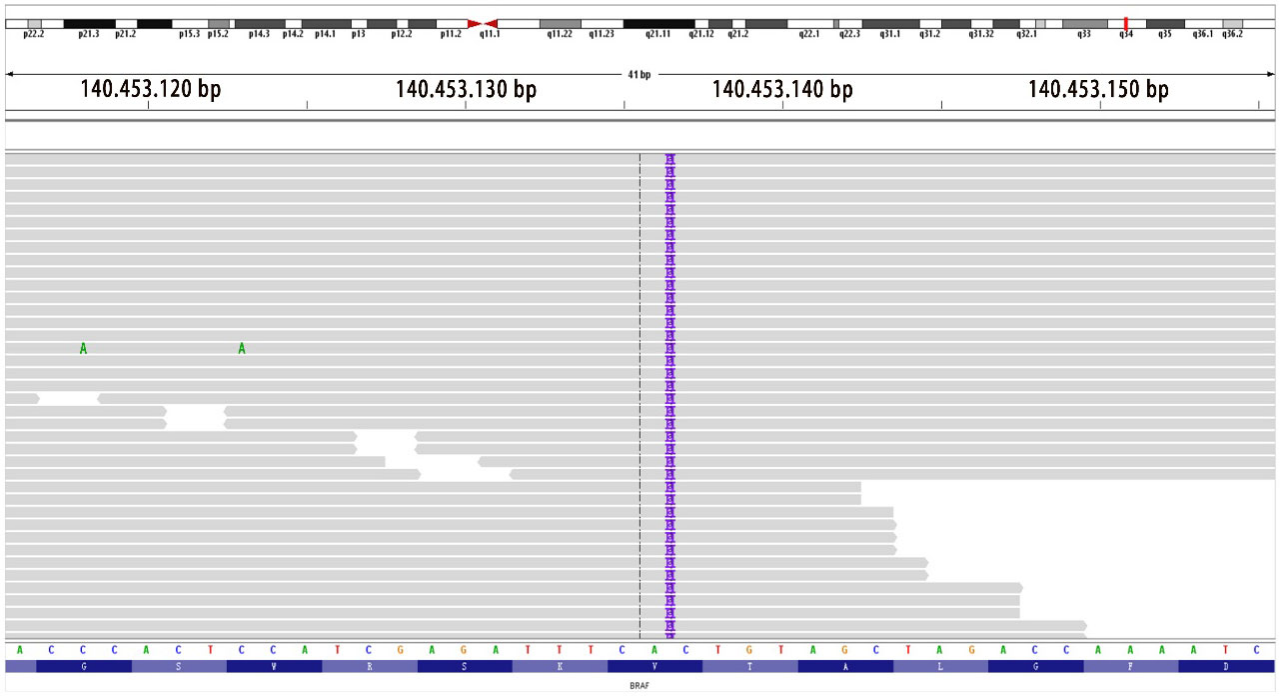
Location mapping of variant sites in the genome and proteins. Mutation type: insertion–deletion mutation; genomic location: NM_004333 exon15 g.140453136 insAAT and c.1798_1799insATT p.V600delinsDL (variant allele frequency, VAF 26.1%).

A male infant aged 5 months 18 days presented with non-bilious greenish vomiting for 4 days without an obvious trigger. Other symptoms included fever (maximum temperature, 37.8°C) and three episodes of loose green stools per day. Abdominal ultrasound ruled out hypertrophic pyloric stenosis. Physical examination revealed abdominal distention, muscle tension, and tenderness. Preoperative findings indicated leukocytosis 15.7 × 10^9^/L (normal range, 5.6–11.4 × 10^9^/L) and elevated hypersensitive C-reactive protein 120.02 mg/L (normal range, <10 mg/L). Preoperative abdominal ultrasound (**Figure 2**) showed the following findings: lower abdomen showing dilated intestines with poor peristalsis; an abnormal echo mass in the right upper abdomen, approximately 31 × 19 mm in size with unclear borders and uneven internal echogenicity, surrounded by enhanced mesenteric echoes; another hypoechoic mass in the right middle lower abdomen measuring 27 × 23 mm, with clear borders and uniform internal echogenicity; and peripheral blood flow signals visible. The diagnosis was suggestive of small bowel high-level incomplete obstruction, with a suspected mass in the right upper abdomen, possibly bowel swelling, torsion, or another mass. Abdominal X-ray (**Figure 3**) showed intestinal gas accumulation, but no significant bowel dilation was noted, and multiple gas-fluid levels were seen in the middle upper abdomen. These findings were suggestive of incomplete small bowel obstruction. After admission, the patient was treated with cefoperazone sulbactam for infection, along with fasting, gastrointestinal decompression, antispasmodics (e.g., mesalazine), probiotics to adjust the intestinal flora, and supportive fluid therapy. However, the symptoms did not resolve, and urgent surgery was performed. Upon exploration, a large amount of turbid ascitic fluid was observed, and a small intestinal mass, approximately 16 cm in size, was found protruding into the lumen (**Figure 4**). The mass was adherent to the right iliac fossa, and after separating the adhesions, marked dilation of the proximal small intestine was noted. The mass was perforated through the intestinal wall, but the tumor capsule was intact, and the surrounding intestinal wall showed scattered nodular growths. The mass was resected, and the specimen was sent for pathological examination. Postoperative recovery was stable, with good wound healing and restored gastrointestinal peristalsis, and the patient was discharged on postoperative day 9. The surgical pathology report revealed that the tumor, lacking a capsule, infiltrated the entire bowel wall and extended into the serosal fat tissue, affecting the mucosa. The tumor cells were spindle-shaped, round, or oval and arranged in bundles or nests. Some areas showed vascular dilation, with tumor cells surrounding blood vessels, resembling angiofibromatosis. Mitosis was rare, and necrosis was not observed. Some areas exhibited lymphocytic infiltration. Immunohistochemistry (**Figure 5**) showed that the tumor cells were positive for S-100 (focal +), smooth muscle actin (SMA) (partially +), CD10 (partially +) (**Figure 5C**), CD56 (partially +), P53 (hotspot, approximately 20%), Ki-67 (hotspot, approximately 30%), CD99 (weak +), SOX10 (−), caldesmon (−), CD34 (−), CD117 (−), ALK (−), CK (−), β-catenin (−), NTRK (−), NKX2.2 (−), DOG-1 (−), desmin (−), ALK(D5F3) (−), negative for ALK (D5F3)-Neg, negative for AE1/AE3, negative for CK8/18, partial (+) for cyclin D1 (**Figure 5D**), negative for DDIT3, negative for desmin, negative for DOG1, negative for D2-40, negative for GLI1, negative for MDM2, negative for MSA, negative for SMA, and negative for STAT6. Molecular testing using panoramic DNA sequencing and PLUS DNA next-generation sequencing (NGS) detected the BRAF p.V600delinsDL mutation (**Figure 6**). The lesion was diagnosed as a low-grade malignant spindle cell tumor of the small intestine, with some tumor cells showing myofibroblastic differentiation.

### 1.2 Discussion

IFS is a rare mesenchymal tumor in early infancy, belonging to non-striated muscle soft tissue tumors (5). It typically presents as a rapidly growing, ill-defined, painless mass with a low metastatic rate (6). It most commonly affects the limbs, trunk, and head/neck and is rarely found in the gastrointestinal tract (7). The condition is more common in male than female patients (8), with a good prognosis and overall survival rates of 90%–100% (9). Surgical resection of the tumor is the preferred treatment. Surgical approaches include open abdominal resection with intestinal anastomosis, laparoscopic exploration with bowel resection and anastomosis, or tumor resection with stoma formation. If the tumor cannot be completely excised, neoadjuvant chemotherapy may be used to shrink the tumor before surgery. Common chemotherapy regimens include the VAC regimen (vincristine, actinomycin D, and cyclophosphamide) and the AVCP regimen (adriamycin, vincristine, cyclophosphamide, and cisplatin). For patients who cannot undergo surgery or have visible residual disease, chemotherapy is recommended as the first-line treatment (9). In this case, after complete resection of the tumor, follow-up through outpatient and online consultations for feeding status, vomiting, and bowel movements showed no recurrence or metastasis at 8 months. The blood routine showed no anemia, and abdominal ultrasound confirmed no recurrence.

Previous literature on two cases of BRAF gene mutation and gastrointestinal disease associated with IFS-like tumors was reviewed: the first case (10) involved a 3-day-old female infant with spontaneous perforation in the mid to distal jejunum, with tumor infiltration into the serosa and surrounding fat tissue. Tumor cells were negative for desmin, SMA, MSA, myogenin, myoD1, β-catenin, CD34, ERG, S100, PHOX2B, NKX2.2, pan-cytokeratin, CD45, CD68, CD117, ALK, CD1a, and CD207, while retaining INI-1 expression. The Ki-67 proliferation index was approximately 1%–2% (+). Genetic analysis revealed a lysine-to-asparagine substitution at the K601 site (K601N). The tumor was completely excised surgically, and the prognosis was favorable. The second case (4) involved a 9-day-old male infant with a rectal mass infiltrating the intestinal wall and partially affecting the mucosa. Immunohistochemical analysis showed diffuse SMA expression, patchy S100 expression, and weak local CD34 positivity. NGS detected the BRAF p.V600D mutation without any additional fusion or point mutations. The patient was subsequently lost to follow-up.

In this case, the patient’s diagnosis, based on histology, immunohistochemistry, and molecular diagnosis, was confirmed after consultation with three hospitals. The detected BRAF p.V600delinsDL mutation (VAF 26.1%) is seen in BRAF-altered spindle cell sarcomas, which can present with features similar to those of IFS, such as oval and spindle cells, infiltrative growth, mucinous stroma, and inflammatory cell infiltration. However, the immunohistochemical and clinical characteristics are not well defined (10). In 2022, Kudo et al. (11) reported the same BRAF mutation, BRAF V600delinsDL (c.1798_1799insATT, p.Val600delinsAspLeu), which was identified as an activating mutation (data not provided). A review of previous literature identified three reported cases with the BRAF p.V600delinsDL mutation. One case was observed in pediatric Langerhans cell histiocytosis (12), and two cases were found in desmoplastic infantile ganglioglioma (13). Tumors harboring BRAF mutations do not necessarily indicate a worse prognosis. Therapeutically, type 2 RAF inhibitors and MEK inhibitors can be considered for treatment.

Most IFS tumors exhibit characteristic t(12;15)(p13;q25) chromosomal translocations, resulting in ETV6–NTRK3 gene rearrangement. While the t(12;15)(p13;q25) translocation is characteristic of IFS, its absence does not rule out the diagnosis of IFS (14, 15). Some studies have suggested that tumors with IFS morphological features may harbor fusions in other genes associated with the mitogen-activated protein kinase (MAPK) pathway, including fusions involving NTRK1/2/3, MET, and RET, or mutations in genes encoding downstream effectors such as RAF1 (CRAF) and BRAF (15), which drive cellular or tumor proliferation and survival. Among these, BRAF mutations are observed in various cancers but are found in less than 0.6% of soft tissue sarcomas (16, 17). BRAF mutations can be categorized into three classes: Class I BRAF mutations involve missense mutations at valine 600 (p.V600), which activate the downstream MAPK pathway independently of upstream RAS activation. These mutations exhibit significantly elevated kinase activity and are commonly found in melanoma, thyroid cancer, and colorectal cancer. Treatment options include BRAF inhibitors or combination therapies with BRAF/MEK inhibitors. Class II BRAF variants include non-p.V600 missense mutations, fusions, and in-frame deletions. These mutations have moderate-to-high kinase activity and are often observed in prostate cancer, bladder cancer, and non-small cell lung cancer. Class III BRAF variants are also non-p.V600 mutations but exhibit low kinase activity. These are frequently identified in cervical cancer, hepatobiliary cancer, and non-small cell lung cancer. The majority (80%) of BRAF mutations occur at the V600 site. Such mutations have also been detected in subsets of gastrointestinal stromal tumors (GISTs) and glomus tumors. Additionally, BRAF gene fusions have been identified in certain inflammatory myofibroblastic tumors, a clinically and morphologically distinct tumor type characterized by alternating myxoid and solid regions typically located in the extremities (18). Therefore, if a tumor lacks the characteristic ETV6–NTRK3 fusion, molecular diagnostics should include testing for abnormalities in other kinases, such as BRAF, NTRK1, and MET (19).

IFS must be differentiated from GISTs, smooth muscle sarcomas, and other spindle cell neoplasms using histological, immunophenotypic, and molecular analyses. Unlike IFS, GISTs are typically characterized by CD117 and DOG1 positivity (20). Leiomyosarcoma, rhabdomyosarcoma, malignant peripheral nerve sheath tumor, and myofibromatosis show positive immunostaining for vimentin, neuron-specific enolase (NSE), estrogen receptor (ER), and desmin. They are negative for cytokeratin, myelin basic protein (MBP), and myoglobin. These tumors can therefore be excluded through the appropriate combination of antibody panels and fluorescence *in situ* hybridization (FISH) analysis (21).

### 1.3 Conclusion

In summary, infantile fibrosarcoma of the intestine is a rare mesenchymal tumor that typically presents in the early neonatal period, with primary manifestations of intestinal perforation and obstruction. Complete surgical resection of the lesion often leads to a favorable prognosis. This report describes the third case of a BRAF gene mutation associated with a gastrointestinal disease in an IFS-like tumor and reviews relevant literature. The aim is to improve understanding and analysis of this tumor, providing diagnostic and treatment insights for early diagnosis and management. Common imaging studies, including ultrasound, CT, and MRI, have limited specificity and diagnostic value, so a definitive diagnosis requires pathological evaluation, supported by histological, immunohistochemical, and molecular diagnostic methods.

## Data Availability

The original contributions presented in the study are included in the article/supplementary material. Further inquiries can be directed to the corresponding author.
